# A SILAC-Based Screen for Methyl-CpG Binding Proteins Identifies RBP-J as a DNA Methylation and Sequence-Specific Binding Protein

**DOI:** 10.1371/journal.pone.0025884

**Published:** 2011-10-03

**Authors:** Stefanie J. J. Bartels, Cornelia G. Spruijt, Arie B. Brinkman, Pascal W. T. C. Jansen, Michiel Vermeulen, Hendrik G. Stunnenberg

**Affiliations:** 1 Department of Molecular Biology, Nijmegen Centre for Molecular Life Sciences, Radboud University Nijmegen, Nijmegen, The Netherlands; 2 Department of Molecular Cancer Research, University Medical Center Utrecht, Utrecht, The Netherlands; Université Paris-Diderot, France

## Abstract

**Background:**

DNA methylation is an epigenetic modification that plays a crucial role in a variety of biological processes. Methylated DNA is specifically bound by Methyl-CpG Binding Proteins (MBPs). Three different types of MBPs have been identified so far: the Methyl-CpG Binding Domain (MBD) family proteins, three BTB/POZ-Zn-finger proteins, and UHRF1. Most of the known MBPs have been identified via homology with the MBD and Zn-finger domains as present in MeCP2 and Kaiso, respectively. It is conceivable that other proteins are capable of recognizing methylated DNA.

**Methodology/Principal Findings:**

For the purpose of identifying novel ‘readers’ we set up a methyl-CpG pull-down assay combined with stable-isotope labeling by amino acids in cell culture (SILAC). In a methyl-CpG pull-down with U937 nuclear extracts, we recovered several known MBPs and almost all subunits of the MBD2/NuRD complex as methylation specific binders, providing proof-of-principle. Interestingly, RBP-J, the transcription factor downstream of Notch receptors, also bound the DNA in a methylation dependent manner. Follow-up pull-downs and electrophoretic mobility shift assays (EMSAs) showed that RBP-J binds methylated DNA in the context of a mutated RBP-J consensus motif.

**Conclusions/Significance:**

The here described SILAC/methyl-CpG pull-down constitutes a new approach to identify potential novel DNAme readers and will advance unraveling of the complete methyl-DNA interactome.

## Introduction

DNA methylation is an epigenetic modification that is essential for a variety of biological processes. In mammals, DNA methylation primarily occurs at cytosines in a CpG dinucleotide context. De novo DNA methyltransferases Dnmt3a and Dnmt3b and maintenance methyltransferase Dnmt1 are responsible for the establishment and maintenance of the DNA methylation mark, respectively. With the exception of CpG-islands, short regions of high CpG-density that are often associated with gene promoters, the mammalian genome is globally methylated [1]. Most CpG-islands remain unmethylated during normal development, whereas aberrant CpG-island hypermethylation is a hallmark of cancer. CpG-island methylation is generally associated with transcriptional repression, and more recently gene-body DNA methylation has been associated with transcriptional activity [2].

Methylated CpGs are specifically bound by methyl-CpG binding proteins, and three families of MBPs are known in mammals (reviewed in [3], [4]). The MBD family proteins MBD1, MBD2, MBD4 and MeCP2, bind methylated DNA via the Methyl-CpG Binding Domain (MBD) [5]. Family members MBD3, MBD5 and MBD6 are, however, incapable of binding methyl-CpG [5], [6]. The BTB/POZ-Zn-finger proteins Kaiso, ZBTB4 and ZBTB38 bind methylated DNA via three C2H2 zinc finger motifs [7], [8]. Most recently, the SET and RING finger-assocoiated domain (SRA), present in UHRF1 and UHRF2, was also identified to specifically recognize methylated DNA [9], [10], [11]. Many reports have shown interactions between MBPs and other proteins or complexes that function in heterochromatinization and transcriptional repression (e.g. [12], [13], [14]), which is thought to account for DNA methylation associated gene silencing.

NMR and crystal structures of MBD and SRA proteins in complex with methylated DNA have shed light on the modes of methyl-CpG binding, which are completely different for the two types of domains [15], [16], [17]. Methylated DNA binding by Kaiso family members may occur via the canonical DNA binding mechanism of C2H2 zinc-finger proteins, as has been suggested in [18], however, the DNA binding domain structure of these proteins has not been resolved yet. Whereas UHRF1 and the Kaiso-related proteins have high affinity for hemimethylated DNA [15], [18], the MBD fold binds symmetrically methylated DNA [17]. For several MBPs sequence context dependent binding to methylated DNA has been described. MeCP2 prefers methylated sites flanked by A/T tracts, which in part is explained by tightening of the minor groove by these tracts [16], [19]. The Kaiso-family members and MBD1 bind methylated DNA in a sequence-specific manner only, and the nucleotides surrounding the methyl-cytosine directly contribute to binding affinity [18], [20]. With the recent discovery of hydroxymethyl-cytosine in genomic DNA of certain tissues, the question raised whether this modification is also recognized by MBPs. It was shown before that hydroxylation of methyl-cytosine interferes with binding of the MBD of MeCP2 [21], and in accordance recently it was reported that full-length MBD1, MBD2 and MBD4 do not bind sequences containing hydroxymethyl-cytosine [22]. In contrast, the SRA domain of UHRF1 binds methylated and hydroxymethylated DNA with similar affinity [23].

The earliest studies on identification of MBPs were performed two decades ago and describe the detection of methyl-DNA binding activities in nuclear extracts by EMSAs and Southwestern assays [24], [25]. MeCP2 was the first MBP that was subsequently purified by traditional biochemical approaches [25]. The definition of the MBD domain of MeCP2 [26], led to the identification of the other MBD family members via homology searches for this domain [5], [27]. Similarly, ZBTB4 and ZBTB38 were identified via homology searches for Kaiso-like Zn-fingers [8], after characterization of Kaiso [7]. The latter was, like MeCP2, initially discovered as a methyl-DNA binding activity in nuclear extracts and biochemically purified. The last decade major developments in mass-spectrometry-based proteomics have taken place, enabling the identification and quantification of thousands of proteins in complex mixtures. High-throughput interactomics approaches are now also starting to be applied in methyl-DNA interactome research. In a recent study, SILAC-based screening was performed to identify proteins whose binding to nucleosomes is regulated by methylation of DNA and/or histones [28]. Nucleosome interacting proteins were purified from SILAC-labeled nuclear extracts and enrichment on modified versus unmodified DNA/nucleosomes was quantitatively determined in mass spectrometry. The results revealed many proteins and complexes that can read the chromatin modification status.

We here follow a similar SILAC-based approach for the identification of potential novel methyl-DNA binding proteins. As outlined above, most of the known DNAme readers have been identified via homology searches. Other DNAme readers may exist that were thus far unidentified due to the lack of unbiased screening methodology. It is conceivable that ‘novel’ protein folds, as well as known DNA binding motifs, may allow for recognition of methylated DNA. Indeed, an example of the latter phenomenon is the Kaiso-family, that binds methylated DNA via canonical C2H2 zinc-fingers. Thus, possibly other known DNA binding motifs bind methylated DNA in sequence context dependent or independent ways. We here describe a screening method to explore the methyl-DNA interactome, by combining SILAC labeling and quantitative mass spectrometry with methyl-CpG pull-downs. We show the results of a methyl-CpG pull-down/SILAC experiment with U937 nuclear extracts and DNA oligos based on a human CpG-island. Among various known DNAme readers we uncovered RBP-J as a methylation dependent binder to the DNA used in the pull-down. Follow-up experiments showed that RBP-J binds to methylated DNA in a sequence specific manner, namely in the context of a mutated consensus sequence.

## Results

### A SILAC-based assay to identify novel methyl-CpG binding proteins

**Figure 1:**
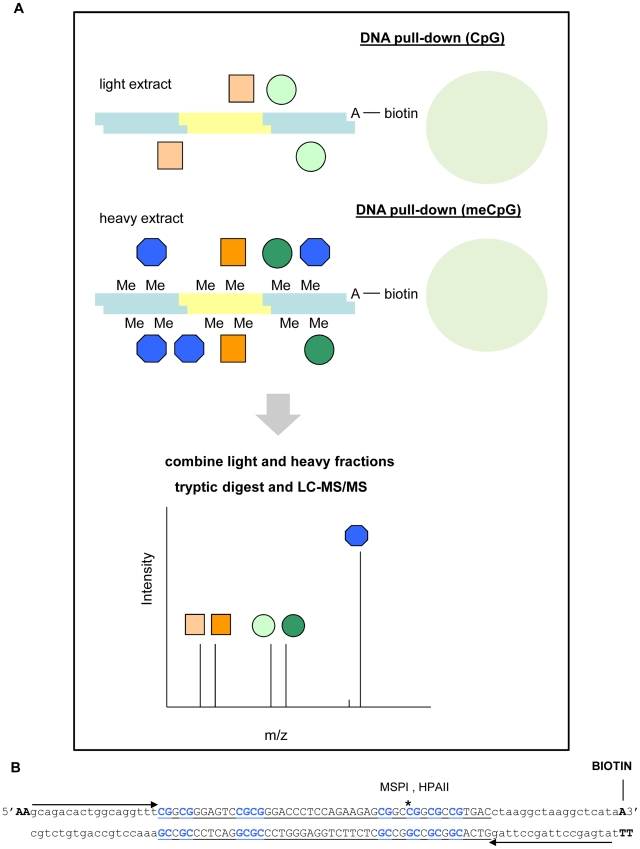
A SILAC-based approach to identify novel methyl-CpG binding proteins. (A) Schematic depiction of the methyl-CpG pull-down assay combined with SILAC. Synthetic DNA is coupled to beads to capture binding proteins from a nuclear extract. Shown is a forward experiment, in which fully methylated DNA is used in a heavy-labeled extract and unmethylated DNA in an unlabeled extract. In a reverse experiment unmethylated DNA is used in a heavy-labeled extract, whereas methylated DNA is used in an unlabeled extract. After several washing steps and elution, both pull-down fractions are combined and analysed by mass spectrometry. Proteins that directly or indirectly specifically bind to the methylated DNA are identified by the quantitative ratios between heavy and light form. (B) DNA used in the methyl-CpG pull-down. The DNA fragment contains part of the sequence of the *GSTP1* CpG-island, sites for primer annealing and a methylation-sensitive restriction site. After ligation a mixture of fragments with different lengths is obtained, which is subsequently biotinylated and methylated. Methylation is checked by a methylation-sensitive digestion followed by quantitative PCR.

To identify novel DNAme readers, we set up a methyl-CpG pull-down assay combined with SILAC, schematically depicted in Fig. 1A. In this assay, proteins are captured from a protein extract by DNA coupled to beads, and quantitatively analyzed by mass spectrometry. In a forward experiment, fully methylated synthetic DNA is used as a bait for MBPs in a heavy-labeled nuclear extract, whereas unmethylated DNA is used in a light (normal) extract. In a reverse experiment, methylated DNA is used in a light extract and unmethylated DNA in a heavy extract. Both pull-down fractions (heavy and light) are mixed, and proteins that directly or indirectly specifically bind to methylated DNA *in vitro* will be identified by high heavy/light ratios in the forward experiment and low heavy/light ratios in the reverse experiment in subsequent mass spectrometry analyses.

**Figure 2:**
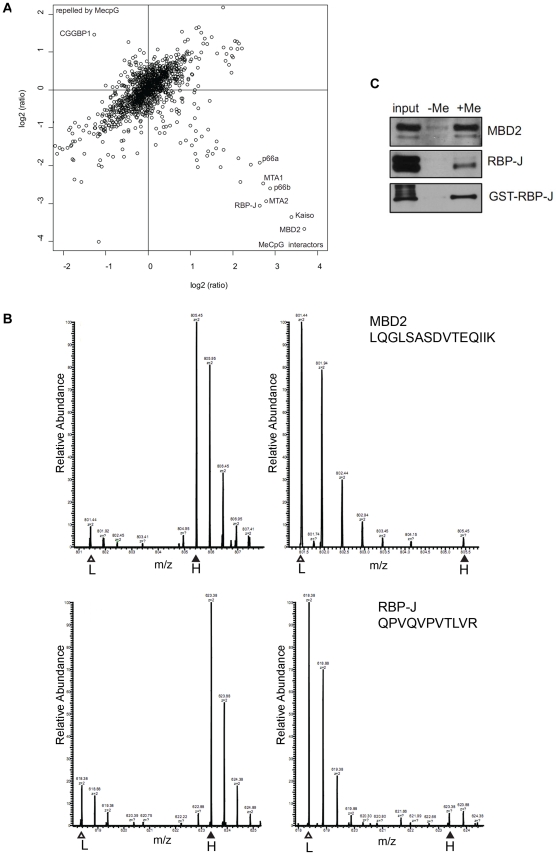
RBP-J preferentially binds a methylated CpG-island sequence *in vitro*. (A) Results of a methyl-CpG pull-down/SILAC experiment with U937 nuclear extracts. Forward and reverse pull-downs were performed, and forward heavy/light ratios of identified proteins were plotted against their reverse heavy/light ratios in a scatter-plot. Proteins that bind specifically to the methylated DNA show high ratios in the forward experiment and low ratios in the reverse experiment and therefore cluster in the lower right quadrant. Background binders appear around the centre of the axes with ratios close to one in both experiments. (B) RBP-J specifically binds to the methylated *GSTP1* CpG-island DNA. Shown are MS signals of peptides from MBD2 (upper panel) and RBP-J (lower panel) from both forward (left) and reverse (right) experiments. L, light; H, heavy. (C) RBP-J directly binds to the methylated *GSTP1* CpG-island DNA. Methyl-CpG pull-down experiments were performed, using western blotting as a read-out. Upper and middle panel: pull-down with U937 nuclear extract and probing for MBD2 (positive control) and RBP-J. Lower panel: pull-down with cleared lysate of E. coli expressing GST-tagged human RBP-J and probing for RBP-J.

We performed a methyl-CpG pull-down with U937 nuclear extracts in forward and reverse using oligos containing part of the sequence of the human *GSTP1* CpG-island (Fig. 1B); its hypermethylation is characteristic in prostate cancer [29]. Proteins that showed at least 1.5 fold enrichment or exclusion on methylated DNA in both forward and reverse experiments are presented in Table S1. A list of all identified proteins with accompanying forward and reverse heavy/light ratios is given in Table S4. To visualize the results, we generated a scatter-plot of forward and reverse heavy/light ratios of all identified proteins (Fig. 2A). Proteins that specifically bind to methylated DNA cluster in the lower right quadrant, whereas proteins that are repelled by methylation of the DNA reside in the higher left quadrant of the scatter-plot. MBD2 was most strongly enriched on methylated oligonucleotides and showed forward and reverse ratios of 12.89 and 0.08, respectively. Moreover, we identified the known DNAme readers Kaiso, MeCP2, UHRF1 and MBD4, as well as almost all subunits of the MBD2/NuRD complex in the lower right quadrant of the scatter plot with ratios of enrichment higher than 1.5 (Fig. 2A, Table S1), indicating that they are methyl-specific binders to our DNA. These results provide proof of principle of our approach and also show the usefulness of the method in the elucidation of protein complexes. MBD3, which does not bind specifically to methylated DNA [5], [13], was identified with forward and reverse ratios close to one (Table S4), which indicated background binding. MBD1, that has both MBD and CXXC domains, of which the first shows sequence specificity [20], was not identified in our experiment. This may be related to low MBD1 protein levels in U937 cells [30]. CGGBP1 was the only protein that showed an exclusion ratio above 1.5 in forward and reverse (Fig. 2A, Table S1), and was therefore identified as repelled by DNA methylation. Indeed, it has been described to bind specifically nonmethylated, but not methylated, 5′-(CGG)(n)-3′ repeats in the promoter of the fragile X mental retardation gene [31], [32]. Altogether, the results demonstrate that our approach successfully identifies proteins and protein complexes recruited or repelled by DNA methylation.

Interestingly, RBP-J/CBF1, the primary mediator of Notch signaling, was found to specifically bind the methylated DNA, as it displayed a high ratio in the forward pull-down and a low ratio in the reverse pull-down (Fig. 2B). The RBP-J interacting protein SPEN was identified in the experiment as well (Table S1 and Fig. S1). To confirm our findings using a different read-out instead of mass spectrometry, we repeated the pull-downs with methylated and unmethylated DNA followed by western blotting with MBD2 (positive control) and RBP-J specific antibodies. Again, recruitment of MBD2 and RBP-J to the methylated DNA but not to the unmethylated DNA was observed (Fig. 2C, upper and middle panel). To address whether RBP-J binds directly to the methylated DNA, cleared lysates of E. coli expressing GST-tagged full-length human RBP-J [33] were used as input for the pull-downs. In the absence of potential human interacting proteins, GST-RBP-J was specifically recruited to the methylated DNA (Fig. 2C, lower panel). Thus, RBP-J binds directly and specifically to the methylated DNA.

### RBP-J is identified as a sequence context specific methyl-CpG binding protein

To further explore DNA methylation dependent binding of RBP-J we performed electrophoretic mobility shift assays. Two different DNA oligonucleotides were used: the GSTP1 oligonucleotide used in the methyl-CpG pull-downs and an oligonucleotide termed GAM12 that contains 12 consecutive 5′-(CAG)-3′ repeats [25]. GST-tagged full-length human MBD2b was used as a positive control for DNAme dependent binding [34]. MBD2b contains the MBD domain, but lacks a 149-amino acid N-terminal domain containing the glycine-arginine rich region in comparison with MBD2a. GST-tagged full-length human RBP-J [33] was used for investigation of RBP-J binding to methylated DNA. RBP-J comprises three structurally integrated domains; the amino (NTD) and carboxy (CTD) terminal domains and a beta-trefoil domain (BTD) inserted in between them [35]. The NTD and BTD cooperate in DNA binding by specific interactions with base pairs in the major and minor grooves and formation of a positively charged surface that interacts non-specifically with the DNA backbone [35].

**Figure 3:**
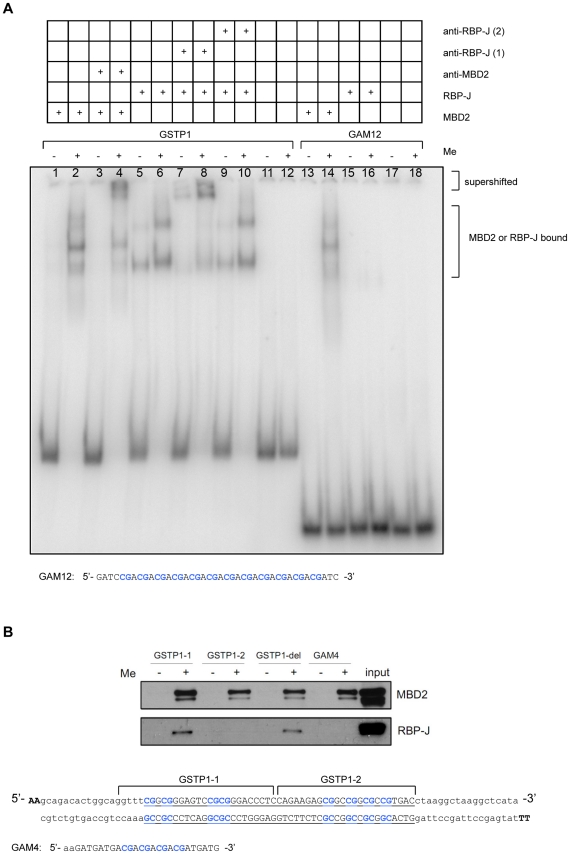
Preferential binding of RBP-J to methylated DNA is sequence specific. (A) RBP-J binds methylated *GSTP1* CpG-island DNA but not methylated GAM12. EMSAs were performed with recombinant GST-tagged human RBP-J, and DNA fragments as indicated. Recombinant MBD2 was used as a positive control. Anti-RBP-J and anti-MBD2 antibodies were added to supershift the DNA. Anti-RBP-J(1): ab25949 (Abcam); anti-RBP-J(2): ab33065 (Abcam); anti-MBD2: 07-198 (Millipore). (B) RBP-J binds to the *first* part of the methylated *GSTP1* CpG-island DNA and not to methylated GAM4. Methyl-CpG pull-downs were performed with U937 nuclear extract and different DNA fragments as indicated. In GSTP1-del the double CpG is replaced by a single CpG. Western blotting was used as a read-out, and MBD2 was probed as a positive control.

Purified recombinant MBD2b and RBP-J were tested for DNA binding. As expected, both MBD2b and RBP-J were able to shift the methylated GSTP1 DNA (Fig. 3A, lane 1–2 and 5–6). For RBP-J, also binding to the unmethylated GSTP1 DNA was observed, however, this binding was much weaker as compared to binding to the methylated DNA. Multiple molecules of either MBD2b or RBP-J bound to the methylated GSTP1 DNA sequence, as multiple shifts were visible per lane. Antibodies against MBD2 and RBP-J were used to supershift the complexes (lane 3–4, 7–8 and 9–10). Only one of two different antibodies against RBP-J was able to supershift (lane 7–8), presumably related to recognition of the native protein. Thus, our EMSA results demonstrated direct and specific binding of RBP-J to methylated DNA.

Surprisingly, MBD2b but not RBP-J bound to methylated GAM12 DNA (lane 13–14 and 15–16). MBD2 is known to bind methyl-CpG via the MBD domain largely independent of the surrounding DNA sequence composition. RBP-J evidently does not bind methylated DNA irrespective of DNA sequence. Therefore, we set out to determine the binding sequence of RBP-J in the GSTP1 DNA. Methyl-CpG pull-downs were performed with DNA corresponding to the first half and second half of GSTP1 (Fig. 3B). Also, DNA corresponding to the first half and containing a single instead of double CpG was tested, as well as oligonucleotide GAM4, containing 4 consecutive 5′-(CAG)-3′ repeats. RBP-J only bound to the first part of GSTP1 DNA, and binding did not depend on the presence of a double CpG (Fig. 3B). Thus, the methylation dependent RBP-J binding site is present in the first half of the GSTP1 DNA. Our results show that RBP-J is a sequence context dependent DNAme binding protein.

### RBP-J binding to a mutated consensus is methylation dependent

**Figure 4:**
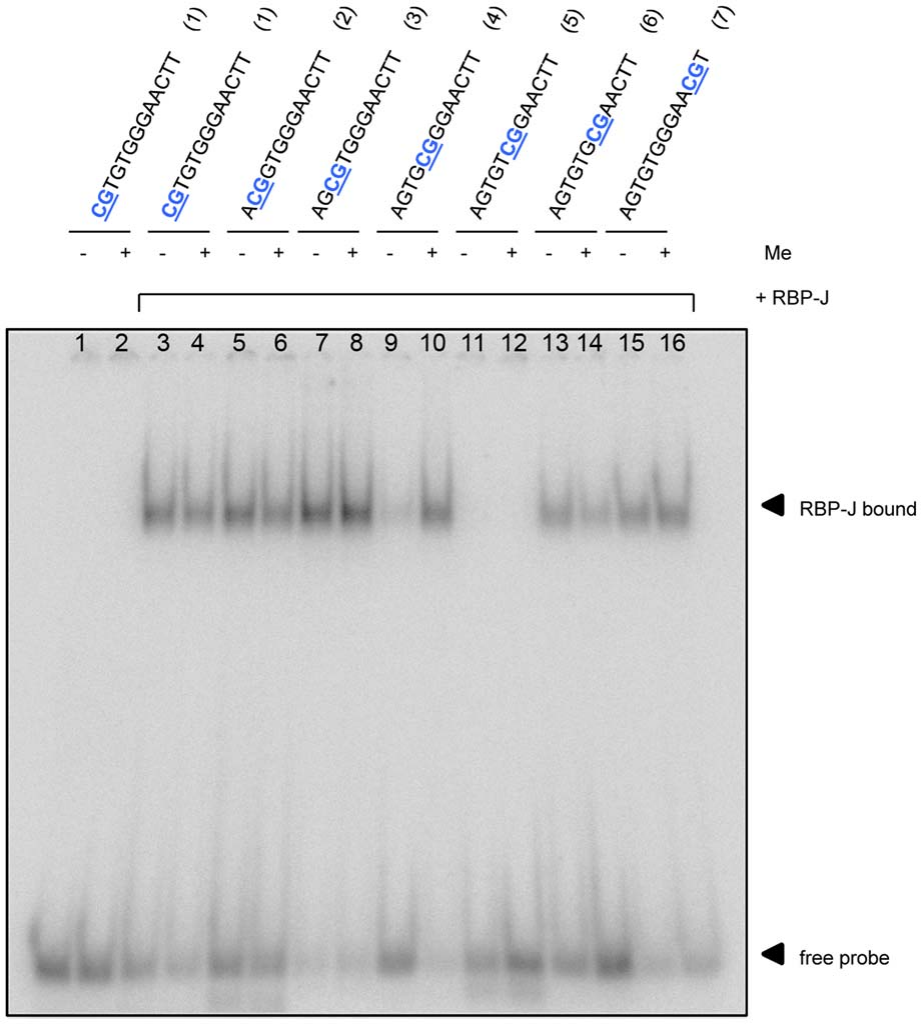
RBP-J binding to a mutated RBP-J consensus site is restored by methylation *in vitro*. RBP-J binding to the perfect RBP-J consensus motif and various altered sites in unmethylated and fully methylated states. EMSAs were performed with recombinant GST-tagged human RBP-J, and DNA fragments as indicated. Altered motifs were designed by substituting residues in the normal consensus (5′-AGCGTGGGAACTT-3′) upstream of a guanine with a cytosine, and residues downstream of a cytosine with a guanine. When required, the single CpG site in the normal consensus was replaced by a TpG to maintain only one CpG per sequence. The resulting 6 altered sites with one CpG per sequence and the normal consensus (lane 7–8) sequence are indicated above lanes.

RBP-J is the main mediator of Notch signaling (reviewed in [36]), and is an extensively studied transcription factor. The DNA binding consensus has been well described. One of the first reports on RBP-J determined the consensus motif by a combination of approaches, including enrichment of RBP-J bound oligonucleotides from a pool of random oligonucleotides [37]. The consensus sequence was established as 5′-ag/ccGTGGGAActa/t-3′, of which the middle hepta-nucleotide sequence is the core recognition motif. We then asked whether and how the observed methylation dependent binding of RBP-J relates to the known consensus motif. For that purpose we designed a CpG-scan throughout the consensus motif; at every guanine or cytosine in the motif, a CpG was created by replacing the upstream or the downstream base by a cytosine and a guanine, respectively. The resulting 6 mutated motifs and the consensus were tested for RBP-J binding in EMSAs in unmethylated and methylated states (indicated in Fig. 4, lane 7–8 contain the consensus).

**Figure 5:**
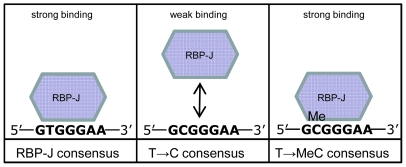
Schematic overview of RBP-J binding motifs *in vitro*. RBP-J is known to bind to the RBP-J consensus motif. Binding is maintained when replacing thymine by a methyl-cytosine, whereas replacement by cytosine results in very weak binding only.

DNA containing the consensus was bound by RBP-J and methylation did not affect binding (Fig. 4, lane 7–8). The CpG creating mutations weakened binding to various degrees; two motifs almost completely lost RBP-J binding (lane 9 and lane 11). Importantly, RBP-J binding to motif 5′-GCGGGAA-3′ was greatly increased by CpG methylation (Fig. 4 lane 9 and lane 10, Fig. S2), whereas binding to other mutated motifs was not affected by methylation. Thus, replacing the thymine preceding the triple guanine by methyl-cytosine in the RBP-J consensus creates a strong RBP-J binding motif. The thymine residue is essential for RBP-J binding, however, a methylated cytosine can functionally substitute for the thymine. Indeed, the same motif that was identified as a methylation dependent binding site for RBP-J in our CpG-scan experiment, is present twice in the first half of the GSTP1 sequence (5′-GCGGGA-3′ at position 3 and 13). Our observations are summarized in Fig. 5. The RBP-J binding motif is well described and the core sequence is 5′-GTGGGAA-3′. Substitution of the thymine by cytosine in the consensus abolishes RBP-J binding, however, methylation of this cytosine restores binding. Presumably, the structural resemblance of thymine and methyl-cytosine allows for interchangeability of these residues, and the mode of binding by RBP-J is similar.

## Discussion

Here we report the application of a methyl-CpG pull-down assay combined with SILAC as a new approach to identify potential novel DNAme readers. Using a DNA sequence that is based on the *GSTP1* CpG-island and U937 nuclear extracts, we recovered several known MBPs and almost all subunits of the MBD2/NuRD complex as methylation specific binders. Moreover, we found RBP-J to bind to this DNA in a methylation dependent way. Follow-up pull-downs and EMSA experiments showed that RBP-J binding to methylated DNA is sequence context dependent. RBP-J binds the consensus motif - 5′-GTGGGAA-3′; replacement of thymine by cytosine abrogates binding, however methylation of this cytosine restores binding.

SILAC-based proteomic screening has been used before in the identification of proteins that interact with specific DNA sequences [38], modified peptides and nucleosomes [28], [39]. The implementation of SILAC-based quantitative mass spectrometry allows for sensitive and rapid screening, and generally requires only a single-step capturing or purification prior to measurement (reviewed in [40]). In a recent study, the effects of DNA and histone methylation on the nucleosome interactome were investigated [28]. Unmethylated versus DNA methylated versions of nucleosome positioning elements, assembled into nucleosomes as well as in naked form, were used in pull-downs. Various proteins displayed methylation dependent binding to only one of the nucleosome positioning elements, thus recognizing CpG methylation in a sequence-specific manner. The most prevalent domains present in these proteins were zinc finger domains and homeoboxes. RBP-J was not identified as a methylation dependent binding protein in this study, which presumably can be attributed to the used DNA sequences. Therefore, the usage of various DNA sequences and extracts from diverse cell types is likely to identify a new group of sequence context-specific methylation dependent binding proteins with diverse DNA binding domains.

We hypothesize that methyl-cytosine functionally substitutes the crucial thymine in the RBP-J consensus motif, creating a structurally similar platform for binding. This phenomenon has been described before. Methylated DNA binding protein (MDBP), a protein originally purified from human placenta, was shown to bind DNA in a sequence-specific and DNA methylation dependent manner [41], [42]. Subsequently, methylation independent binding by MDBP *in vitro* was detected to sites that contain thymine residues replacing methyl-cytosine residues [43]. Methylation-dependent binding sites were located in mammalian genes [44], [45] and methylation independent sites were identified in polyoma virus, cytomegalovirus and hepatitis B virus enhancers, and in a c-Myc intron [44], [46], [47], [48], [49], [50]. MDBP was identified to be regulatory factor for X box (RFX)1 [51], and to be part of a family of closely related proteins with similar DNA binding properties [52], [53]. Another example of thymine and methyl-cytosine substitution in DNA binding sites concerns ZBTB4. Recently, site-selection assays (SELEX) and methyl-SELEX were used to identify the preferred binding sites of ZBTB4 on unmethylated DNA and methylated DNA, respectively. It appeared that ZBTB4 binds to the sequence: 5′-CMGCCAT-3′ (M being methyl-cytosine) [18]. The second best binding site has been defined as 5′-CTGCCAT-3′, in which a thymine replaces the methyl-cytosine. Whether interchangeability of thymine and methyl-cytosine in *in vitro* DNA binding assays applies to specific factors and to what extent this phenomenon occurs *in vivo* remains to be investigated.

The mutated RBP-J consensus site, containing a methyl-cytosine instead of a thymine, is a bona-fide RBP-J binding site *in vitro*, and presumably RBP-J binding to this site is structurally identical to binding to the normal consensus. From our EMSA results, for example as shown in Fig. 4, one might speculate that *in vitro* a thymine containing consensus maybe the preferred binding site of RBP-J over a methyl-cytosine containing consensus. As we have not performed EMSAs with cold competitor DNA, we cannot conclude whether thymine or methyl-cytosine containing sites are the better target. An interesting issue is whether the mutated consensus is used by RBP-J *in vivo*. If so, the DNA methylation status of this site determines RBP-J binding and consequently its Notch responsiveness. Such a DNA methylation dependent on/off switch would add a new layer of regulation to Notch signaling. So far, we did not find evidence for methylation dependent binding in genome-wide binding maps of mouse RBP-J in C2C12 cells (unpublished results S.J.J. Bartels and H.G. Stunnenberg). With the generation of more genome-wide binding profiles for RBP-J in various cell types, the contribution of the mutated consensus to RBP-J binding and gene regulation can be assessed. The here described SILAC-based screen enables unraveling of the complete methyl-DNA interactome, and subsequent *in vitro* assays and the generation of *in vivo* genome-wide binding maps will clarify the roles of potential novel DNAme readers. By using such a systematic approach, our understanding of the DNA methylation code and its interpretation by DNAme readers will be greatly increased.

## Materials and Methods

### Cell culture and SILAC labeling

U937 cells (ATCC) were cultured in RPMI medium containing 10% fetal bovine serum and 1% penicillin/streptomycin (Gibco/Invitrogen) at 37°C in 5% CO_2_ atmosphere. For SILAC labeling, RPMI (-Arg, -Lys) medium (Gibco/Invitrogen) containing 10% dialyzed fetal bovine serum (Gibco/Invitrogen) and 1% penicillin/streptomycin was supplemented with either ^13^C_6_^15^N_4_ L-arginine and ^13^C_6_^15^N_2_ L-lysine (Isotec) or non-labeled L-arginine and L-lysine (Sigma). Cells were cultured in SILAC medium for at least 8 doublings to ensure full incorporation of the labeled amino acids.

### Nuclear extracts

The procedure for nuclear extract preparation was derived from [54]. In short, PBS washed cells were resuspended in 2.5 volumes hypotonic buffer (10 mM HEPES pH 7.9, 10 mM KCl, 0.1 mM MgCl_2_, 0.1 mM EDTA pH 8, 10% glycerol, 1 mM DTT, 1 mM PMSF, and complete protease inhibitors (Roche)), incubated for 30 min on ice, and lysed in a Dounce homogenizer (B type pestle). After centrifugation for 20 min at 2000 g, supernatant was removed and pelleted nuclei were washed twice with PBS. Nuclei were then resuspended in 1 volume hypertonic buffer (20 mM HEPES pH 7.9, 420 mM NaCl, 1.5 mM MgCl_2_, 0.1 mM EDTA pH 8, 10% glycerol, 1 mM DTT, 1 mM PMSF, and complete protease inhibitors), rotated for 1 h at 4°C, and centrifuged for 30 min at 100,000 g. The resulting supernatant/nuclear extract was frozen in liquid nitrogen and stored at −80°C.

### Methyl-CpG pull-down assay

Oligos used in pull-down experiments are listed in Table S2. PAGE purified oligos were annealed and phosphorylated. After ligation DNA fragments containing up to ten multimerized oligos were retrieved with lengths up to 600 bp. The fragments were subsequently biotinylated by incorporation of biotin-14-dATP (Invitrogen) at the 3′end of the forward strand using Klenow Fragment (3′-5′exo-) (New England Biolabs), and purified on Illustra NAP10 columns (GE Healthcare). For MeCpG pull-downs, DNA was methylated by M.SssI (New England Biolabs). Methylation of GSTP1 DNA was checked by methylation-sensitive digestion followed by quantitative PCR. For pull-downs followed by mass spectrometry, 75 µl of Dynabeads MyOne Streptavidin C1 (Invitrogen) were incubated with 10 µg of DNA for 1 h at RT in DNA binding buffer (150 mM NaCl, 50 mM Tris pH 8.0, 0.1% NP40). After washing twice in 1 ml DNA binding buffer, the beads with coupled DNA were incubated with 400 µg nuclear extract and 10 µg poly(dI-dC) competitor DNA (Sigma) for 2 h at 4°C in protein binding buffer (150 mM NaCl, 50 mM Tris pH 8.0, 0.25% NP40, 0.5 mM DTT, and complete protease inhibitors –EDTA (Roche)). Beads were washed five times in 1 ml protein binding buffer, and bound proteins were eluted in SDS PAGE loading buffer and processed for mass spec analyses. For western blot analysis, the same procedure was followed using one quarter of materials (beads, DNA, extract) as described above.

### Mass spectrometry

In preparation for mass spectrometry, captured proteins from DNA pull-downs were separated by SDS PAGE and in-gel trypsin digested. Peptides were then extracted, desalted using StageTips [55], and analyzed on an Orbitrap Velos mass spectrometer, essentially as described [56]. Raw data were processed and analyzed using MaxQuant software (version 1.1.1.25) containing the integrated Andromeda search engine [57], [58] and searched against a human decoy IPI database v3.68 using a false discovery rate of 1% at the protein and peptide level.

### Western blotting

Western blotting was performed according to standard procedures. The following antibodies were used: anti-MBD2 (Everest, EB07538), anti-RBP-J (Abcam, Ab25949).

### Recombinant proteins

pETM30-RBP-J (VK91) plasmid and pGEX-5X-1-MBD2b plasmid, for bacterial expression of human His/GST-RBP-J and GST-MBD2b, respectively, have been described before [33], [34]. Expression and GST purification of recombinant proteins were performed as described in [34]. For methyl-CpG pull-down experiments, crude E. coli lysates were used, in which case the purification procedure was followed up to the addition of Glutathione Sepharose beads. For EMSAs, purified proteins were used.

### EMSA

DNA oligos used in EMSAs are listed in Table S3. GAM12 DNA has been described before [25]. PAGE purified oligos were annealed and methylated by M.SssI (New England Biolabs). DNA was subsequently labeled using gamma-32P-ATP and T4 Polynucleotide Kinase (New England Biolabs), and purified on Illustra ProbeQuant G50 columns (GE Healthcare). 0.1 ng DNA was incubated with 100 ng protein in 20 µl binding buffer (20 mM HEPES pH 7.9, 1 mM EDTA, 3 mM MgCl_2_, 10 mM β-mercaptoethanol, 10% glycerol, 0.1% Triton-X100) containing 2 µg BSA and 10 ng poly(dI-dC) for 30 min on ice. For supershifting, 500 ng of the following antibodies were added: anti-MBD2 (Millipore, 07-198), anti-RBP-J (Abcam, Ab25949) and anti-RBP-J (Abcam, Ab33065). DNA-protein mixtures were run on non-denaturing 6% polyacrylamide and gels were analyzed according to standard procedures.

## Supporting Information

Figure S1 **The RBP-J interacting protein SPEN is preferentially recruited to methylated** ***GSTP1*** **CpG-island DNA.** Shown are MS signals of a peptide from SPEN from both forward (left) and reverse (right) experiments. L, light; H, heavy.
(TIF)

Figure S2 **RBP-J binding to a mutated RBP-J consensus site is restored by methylation** ***in vitro*** **.** EMSAs were performed with recombinant GST-tagged human RBP-J and the DNA probe containing a mutated RBP-J consensus site as indicated. Increasing amounts of RBP-J were added to binding reactions.
(TIF)

Table S1 Results methyl-CpG pull-down/SILAC. Shown are proteins that have forward ratio >1.5 and reverse ratio <0.67 (Me-CpG recruited). CGGBP1 is the only protein with forward ratio <0.67 and reverse ratio >1.5 (Me-CpG repelled). RBP-J and SPEN are indicated in bold; MBPs are indicated by *; subunits of MBD2/NuRD are indicated in grey.
(TIF)

Table S2 DNA oligos used in methyl-CpG pull-down experiments. Sequence derived from the GSTP1 CpG-island is indicated in capitals; the methylation-sensitive restriction site is underlined.
(TIF)

Table S3 DNA oligos used in EMSA experiments. In sequence1–7 oligos the (mutated) RBP-J consensus site is indicated in capitals.
(TIF)

Table S4 Results methyl-CpG pull-down/SILAC of all identified proteins.
(XLS)
