# Supplementary figures and images for: A SILAC-Based Screen for Methyl-CpG Binding Proteins Identifies RBP-J as a DNA Methylation and Sequence-Specific Binding Protein
Source: PLoS One. 2011 Oct 3;6(10):e25884. doi: 10.1371/journal.pone.0025884 (PMC3185043; doi:10.1371/journal.pone.0025884)

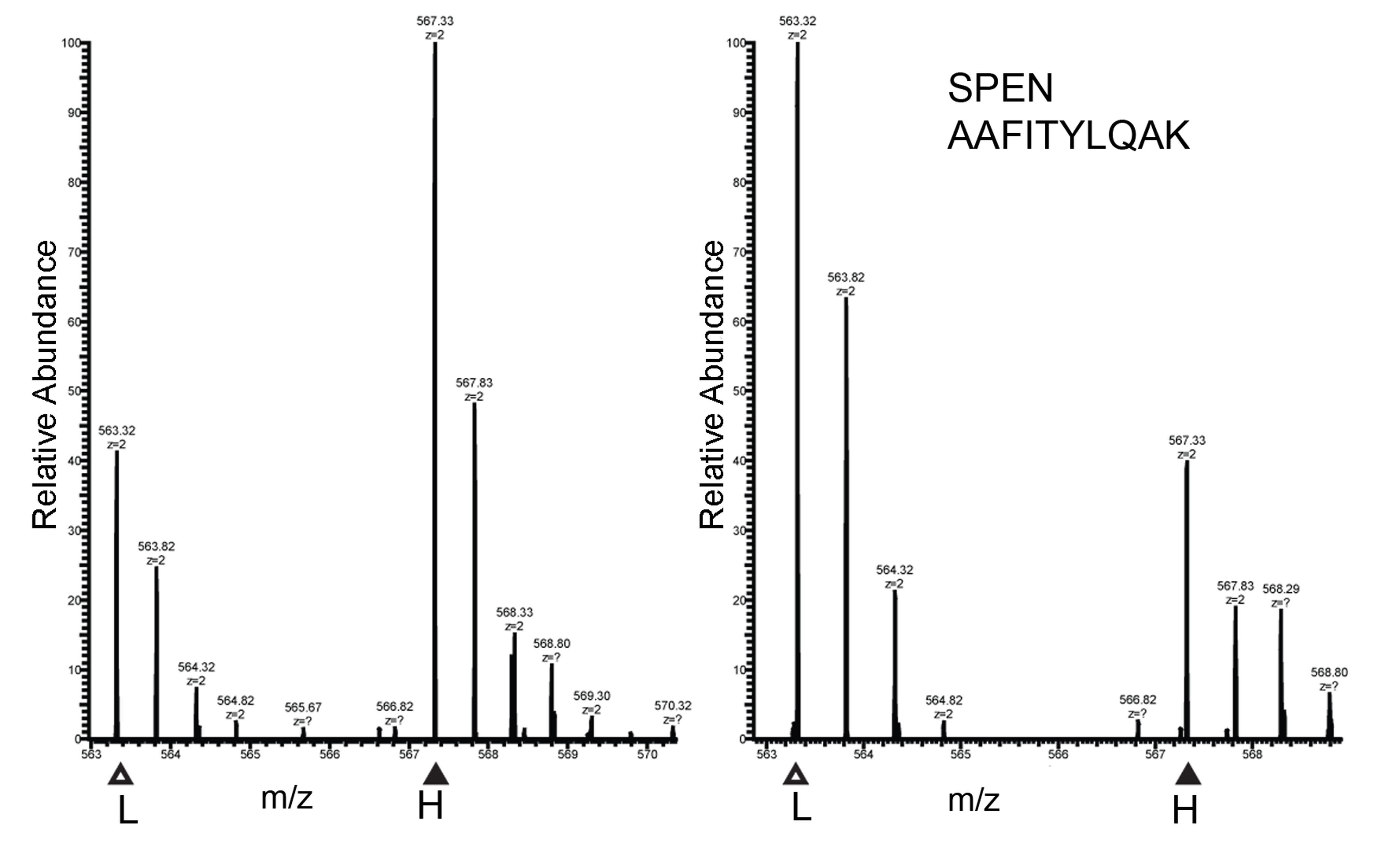

Supplement: Figure S1 — The RBP-J interacting protein SPEN is preferentially recruited to methylated GSTP1 CpG-island DNA. Shown are MS signals of a peptide from SPEN from both forward (left) and reverse (right) experiments. L, light; H, heavy. (TIF) [file pone.0025884.s001.tif]

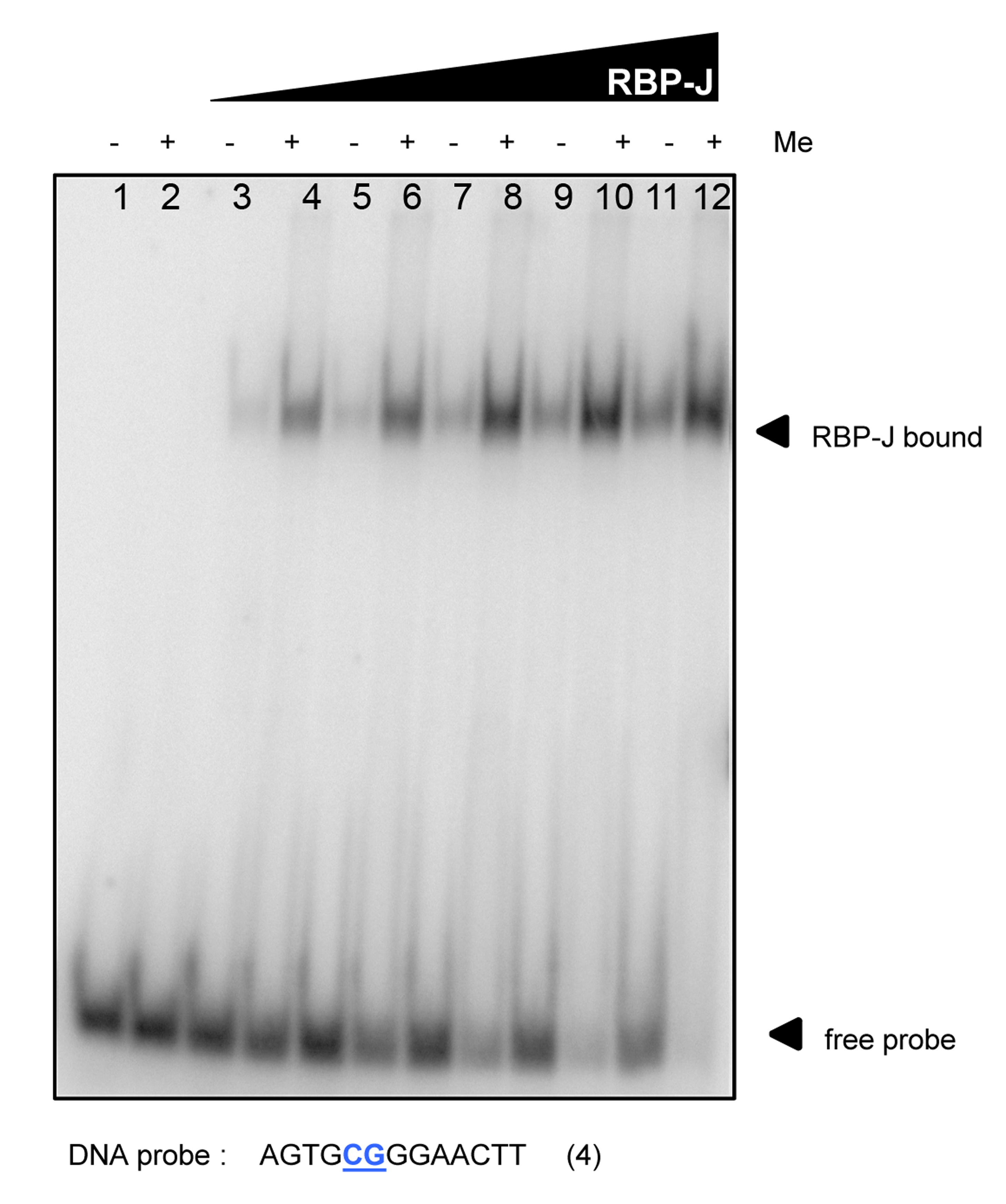

Supplement: Figure S2 — RBP-J binding to a mutated RBP-J consensus site is restored by methylation in vitro . EMSAs were performed with recombinant GST-tagged human RBP-J and the DNA probe containing a mutated RBP-J consensus site as indicated. Increasing amounts of RBP-J were added to binding reactions. (TIF) [file pone.0025884.s002.tif]

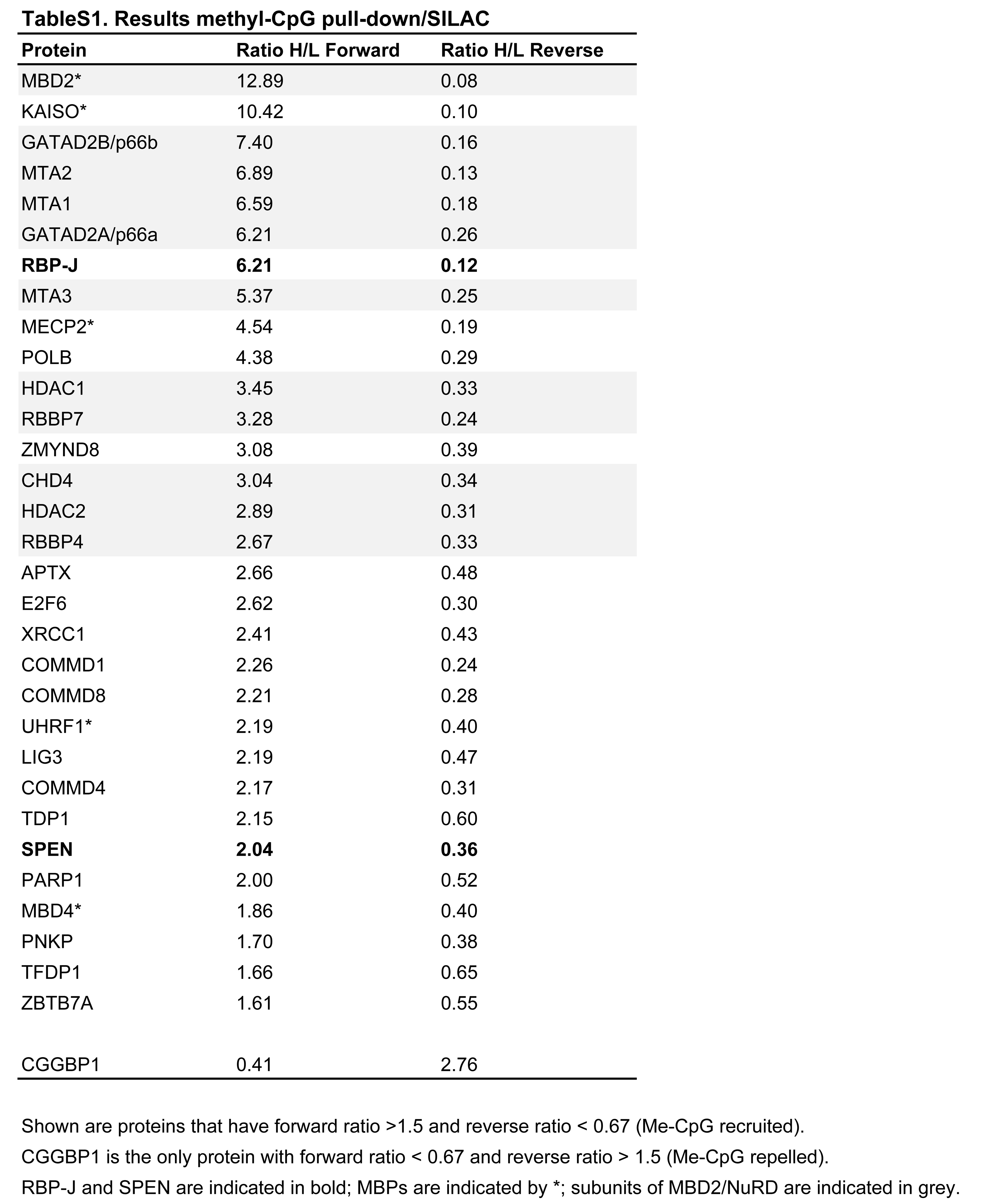

Supplement: Table S1 — Results methyl-CpG pull-down/SILAC. Shown are proteins that have forward ratio >1.5 and reverse ratio <0.67 (Me-CpG recruited). CGGBP1 is the only protein with forward ratio <0.67 and reverse ratio >1.5 (Me-CpG repelled). RBP-J and SPEN are indicated in bold; MBPs are indicated by *; subunits of MBD2/NuRD are indicated in grey. (TIF) [file pone.0025884.s003.tif]

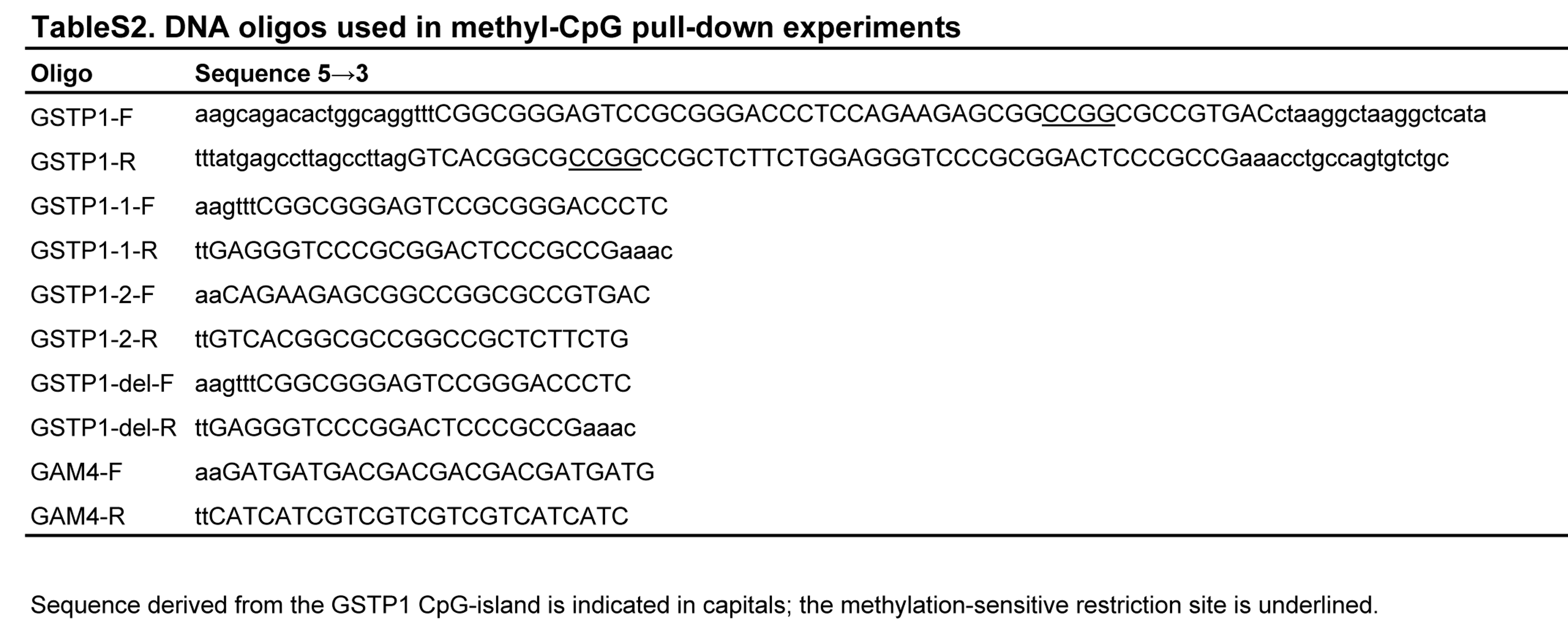

Supplement: Table S2 — DNA oligos used in methyl-CpG pull-down experiments. Sequence derived from the GSTP1 CpG-island is indicated in capitals; the methylation-sensitive restriction site is underlined. (TIF) [file pone.0025884.s004.tif]

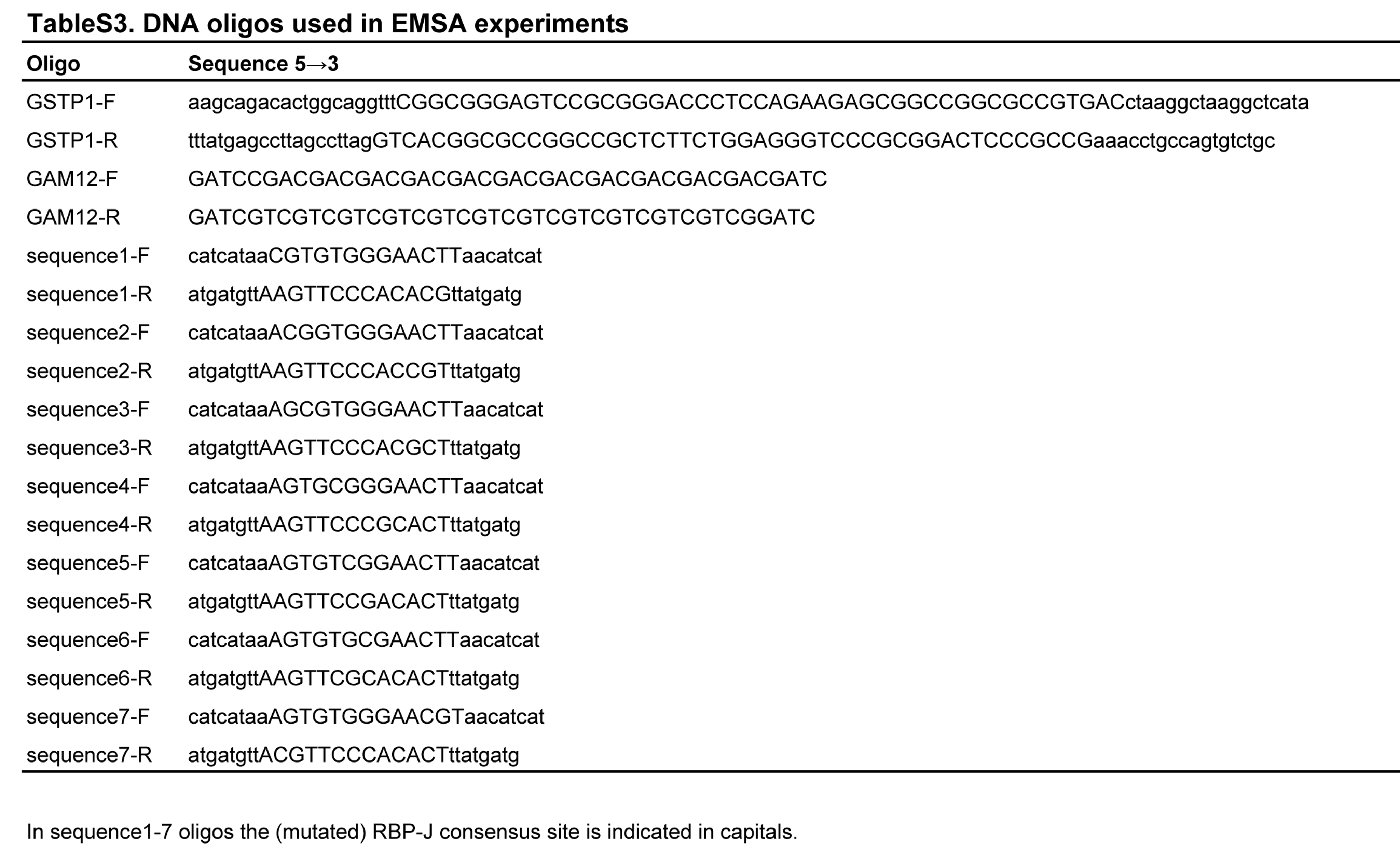

Supplement: Table S3 — DNA oligos used in EMSA experiments. In sequence1–7 oligos the (mutated) RBP-J consensus site is indicated in capitals. (TIF) [file pone.0025884.s005.tif]
